# Geroscience and climate science: Oppositional or complementary?

**DOI:** 10.1111/acel.13890

**Published:** 2023-06-01

**Authors:** Colin Farrelly

**Affiliations:** ^1^ Department of Political Studies Queen's University Kingston Ontario Canada

**Keywords:** ageism, climate change, exposome, geroscience, public policy, science communication

## Abstract

Two of this century's most significant public health challenges are climate change and healthy aging. The future of humanity will be both warmer and older than it is today. Is it socially responsible, in a warming planet of a population exceeding 8 billion people, for science to aspire to develop *gerotherapeutic drugs* that aim to reduce the burden of aging‐related diseases that may also increase lifespan? This question is the “elephant in the room” for geroscience advocacy. Science communication concerning what constitutes empirically valid and morally defensible ways of navigating the dual public health predicaments of climate change and healthy aging must be sensitive to both the *interdependence* of the environment (including planetary health) and the mechanisms of aging, as well as the common (mis)perceptions about the potential conflict between the goals of climate science and geroscience. Geroscience advocacy can transcend narratives of intergenerational conflict by highlighting the shared aspirations of climate science and geroscience, such as the goals of promoting health across the lifespan, redressing health disparities, and improving the economic prospects of current and future generations.

## 1 INTRODUCTION

**Figure 1:**
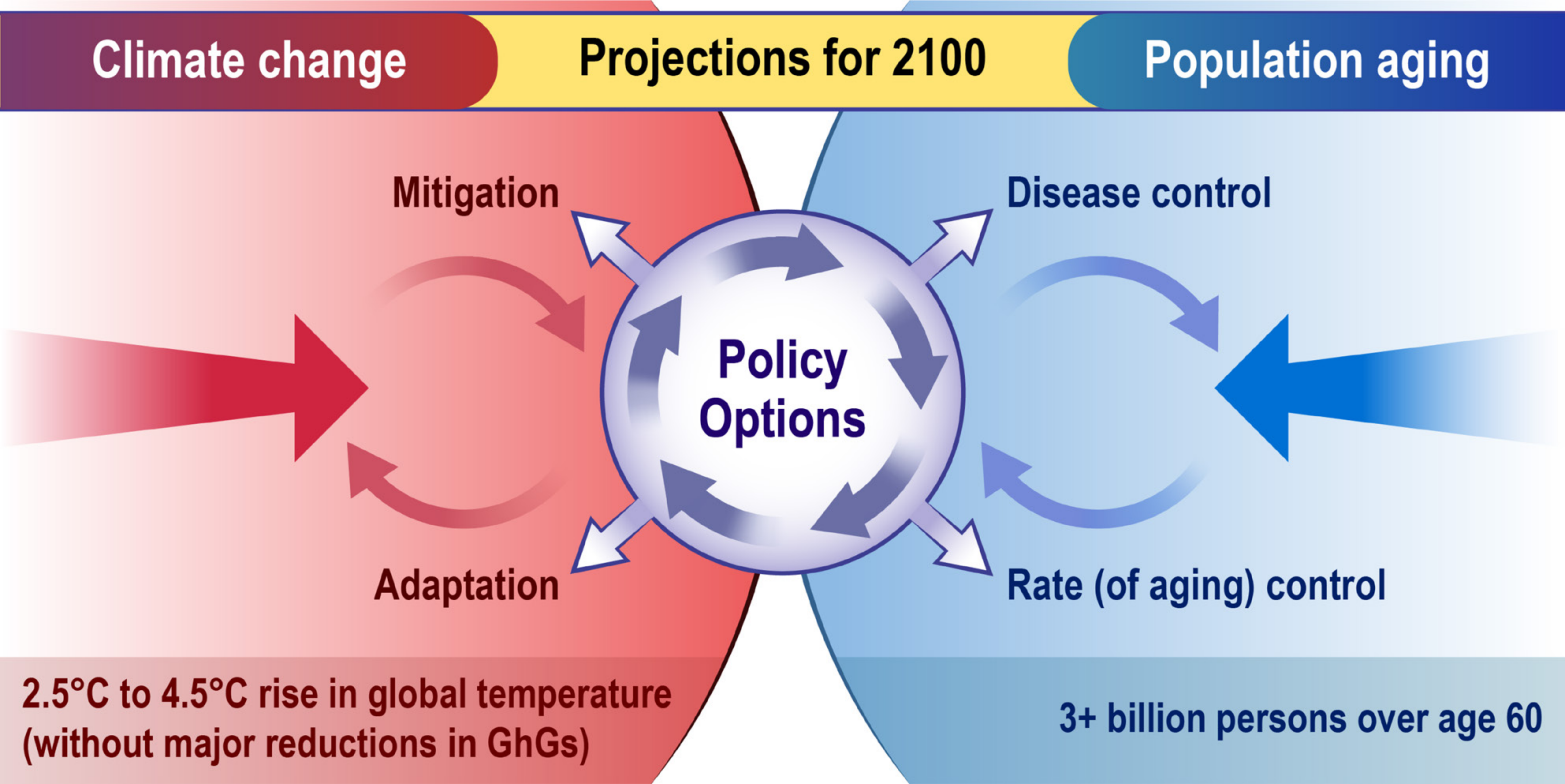
Climate Change in an Aging World.

Two of this century's most significant public health challenges are climate change and healthy aging. The future of humanity will be both warmer and older than it is today. Taken in isolation from each other, tackling either one of the novel public health challenges of climate change or healthy aging requires foresight, scientific innovation, and collaborative governmental action. However, the public health challenges of the 21st century are even more Herculean because climate change and population aging are occurring *simultaneously* (Figure 1). And this means that science communication concerning what constitutes empirically valid and morally defensible ways of navigating these dual public health challenges must be sensitive to both the *interdependence* of the environment and the mechanisms of aging, as well as the common (mis)perceptions about the potential conflict between the goals of climate science and geroscience.

It is a common and accepted role for scientists to get involved in public policy debates, especially if their research pertains to public health (Oppenheimer, 2011). “Responsible biology” entails that scientists conceive of themselves as artisans working for the pubic good, and thus, they have a moral obligation to reflect on the ends (and not just the means) of scientific research (Kitcher, 2004). Is it socially responsible, in a warming planet of a population exceeding 8 billion people, for science to aspire to develop *gerotherapeutic* drugs? That is, drugs that target pathways involved in aging with the aim of reducing the burden of aging‐related diseases and increasing lifespan and healthspan (Le Couteur & Barzilai, 2022). This question is, for the field of geroscience, the “elephant in the room.” It is a question the field must tackle head on vs avoid, lest it risk marginalizing the science of healthy aging.

Unlike scientific innovation for pharmaceuticals treating specific diseases, like cancer, heart disease, or Alzheimer's, biomedical gerontology often faces concerns that arise from what Richard Miller (2002) calls “gerontologiphobia”—“the irrational fear that aging research is a public menace bound to produce a world filled with non‐productive, chronically disabled, unhappy senior citizens consuming more resources than they produce.” Climate anxiety among younger persons, coupled with “egalitarian advocacy” (a motivation to take action and enact equality‐based change), may lead to “succession”‐based ageism—the belief that older adults should step aside to free up coveted opportunities (Martin & North, 2022). The case for shifting public health priorities from the goal of making further increases in lifespan for older populations via disease control toward the goal of increasing the human *healthspan* via rate (of aging) control (Comfort, 1969) can help abate the assumptions of intergenerational conflict underpinning such problematic sentiments.

Rather than conceptualizing the distributional effects of an applied gerontological intervention as something that would only benefit persons in late life (e.g., increasing lifespan), and climate change as something that only imposes health and economic risks primarily on younger generations, attention must be given to the reality that aging and climate change are intricately connected. Not only are older persons at higher risk for climate change mortality, but the health of the environments we inhabit (including planetary health) influence aging and the healthspan. Rate (of aging) control would improve the quality of life of adults at all ages and for future generations versus simply increasing the number of years of survival for the older persons of today. In addition, the economic benefits of slowing aging will better enable populations (especially those in lower income countries) to invest in the adaptations (e.g., changing land and cropping practices; installing better‐draining pavements to deal with floods; improving water storage and use) necessary to minimize some of the harms of climate change.

## 2 CLIMATE CHANGE IN AN AGING WORLD

NASA models (NASA, 2023) estimate that, depending on the action taken to reduce greenhouse gases (GhGs), the global temperature can be expected to rise between 2.5 and 4.5**°**C by the year 2100. This warming is expected to lead to an increase in extreme weather events, heat stress, a diminishment in air quality and threatens continued progress on global food security, etc.

Within this same timeframe, the median age of humans living on the planet will rise from the current age of approximately 31 years to 45.6 years (when not adjusted for longevity increase) (Lutz et al., 2008). This reflects two significant developments. First, improvements in public health and material prosperity have reduced early and mid‐life mortality. The total number of under‐5 deaths worldwide has declined from 12.6 million in 1990 to 5.2 million in 2019 (WHO, 2020). Second, people are having fewer children. By 2100, global fertility is expected to decline below replacement levels (reaching 1.9 by the year 2100 (UN, 2020)) and an estimated 3.1 billion persons will be age 60 or over, including nearly 20% of the population in Africa (the world's youngest continent) (UN, 2023). Global aging is arguably humanity's most significant success story, but it also poses a novel and significant public health predicament—how to realize healthy aging so that older populations can enjoy a better quality of life.

Older persons are more susceptible to many of the health and economic harms of climate change, such as increased respiratory and cardiovascular disease, heat‐related illness and death, and power outages from an increase in extreme weather events. The interconnected public health challenges of climate change and healthy aging present a novel communication predicament for scientists in both fields. If only climate scientists advise and advocate for research on how best to mitigate and adapt to climate change, ignorant of how the biology of aging is implicated in many of the health risks of climate change, then geroscience may be ignored or marginalized or, even worse, portrayed as an aspiration *antithetical* to socially responsible scientific research. Conversely, if biomedical researchers working on modulating the biology of aging are ignorant of the realities of climate change, they may jeopardize their reputation as scientists that take seriously the requirements of responsible biology, and be subject to the criticism that they are “climate deniers.” Interdisciplinary dialogue, understanding and communication is thus imperative.

The data from the World Meteorological Organization's “State of the Global Climate 2020” report show that the global mean temperature for 2020 was around 1.2°C warmer than pre‐industrial times (WMO, 2021). The most direct way in which climate change is expected to affect public health relates to changes in mortality rates associated with exposure to ambient temperature (Hajat et al., 2014). The IPCC 2022 (B 4.4) report contends, with high confidence, that climate change and related extreme events will significantly increase ill health and premature deaths from the near‐to long‐term. The World Health Organization estimates that between 2030 and 2050, climate change is expected to cause approximately 250,000 additional deaths per year from malnutrition, malaria, diarrhea and heat stress alone (WHO, 2023).

The public health risks posed by extreme weather events, heat stress, diminishing air quality, and food supplies will vary for different geographical regions of the world, as well as socio‐economic factors within a population. However, biological factors (e.g., the impact aging has on the risks of multi‐morbidity, frailty, and disability) make older persons and populations among those most vulnerable to the adverse health impacts of climate change (Carnes et al., 2014; Davies & Bhutta, 2022; UN, 2022; Yu et al., 2011). The goal of rate (of aging) control is very distinct from the central public health strategy—“disease control”—deployed over the past century and a half that helped reduce early and mid‐life mortality. Thus the two public health strategies of disease control and rate (of aging) control will have very different impacts on the climate change health risks older persons will likely face by the end of this century.

The early public health pioneer Charles Winslow (1877–1957) detailed, in 1903, the strategy of launching a “war against disease” (Winslow, 1903). Winslow proposed that public health and medicine should tackle each specific infectious disease, one at a time. This same strategy of disease control was later deployed, in the second half of the 20th century, to the degenerative diseases of late life, like cancer (Farrelly, 2021, 2021a) heart disease and stroke. However, despite an annual investment of billions of NIH research dollars over many decades on biomedical research on diseases like cancer, heart disease, and Alzheimer's, not a single disease of aging has been eliminated. Significant progress has been made in postponing death into later life by permitting older persons to *manage* chronic disease, frailty, and disability. Even if a cure for one late onset disease were to be made, such as cancer, that means that more debilitating diseases can become more prevalent because the hazard in old age is not so much that one disease displaces another but that the new diseases are often much more debilitating (Olshansky, 2018). To only pursue the strategy of disease control in a warming and aging world is to expose billions of humans to sub‐optimal health outcomes compared to what could be realized by slowing down the rate of molecular and cellular decline to increase the *healthspan*, thus delaying and compressing the period of time spent living with multi‐morbidity, frailty, and disability in late life. This aspiration is even more critical in a warming world.

## 3 THE INTERPLAY BETWEEN ENVIRONMENT AND BIOLOGY (INCLUDING THE MECHANISMS OF AGING)

Wild (2005) introduced the concept of “exposome”—which encompasses life‐course environmental exposures such as air pollution and also lifestyle factors like smoking—to mirror the precision and attention the medical sciences has given to the human genome in the effort to help prevent and treat disease. Solar UV radiation, for example, accumulates damage, throughout our lifetime, causing inflammation, immune changes, physical changes, as well as the DNA damage that promotes cellular senescence (Amaro‐Ortiz et al., 2014). Noise pollution from modern urban life, such as road, rail, and air traffic noise, affect both objective and subjective assessments of sleep (Basner et al., 2011). The suboptimal diets (e.g., high intake of sodium, low intake of whole grains, and fruits) typically consumed in modern environments are also a major risk factor in non‐communicable diseases (GBD, 2019). There is evidence that obesity accelerates aging, thus shortening the lifespan and healthspan of some obese adults (Salvestrini et al. 2017; Tam et al., 2020). The increasing consumption of ultra‐processed food and the global chain of food production have a negative impact on both human health and planetary health (Avesani et al., 2022).

Features of the exposome can be modulated to improve healthspan, ranging from interventions at the macro‐geophysical environmental level—for example, the design of urban environments more connected to nature‐ to the micro‐tissue and cellular physiology level (Shiels et al., 2021). Non‐pharmacological strategies of improving the healthspan, such as the concept of “food as medicine,” further illuminate the nuanced interdependence between the health of the planet and human health. Spices, for example, are consumed more in the diet of populations living in countries like India than the United States, and have a complex polypharmacology, but, for at least moderate consumption, can help prevent and control many chronic diseases associated to malnutrition from a Western diet (Nilius & Appendino, 2013). Greater blueberry and anthocyanin intake is associated with less weight gain during aging (Bertoia et al., 2015) and numerous clinical studies in neuroscience and blueberries have found their consumption can improve cognitive functioning (Kalt et al. 2020).

One last element of the exposome worth emphasizing to amplify how significant and complex the interdependence between the environment and biological aging is concerns the impact socioeconomic factors can have on both the harms of climate change and senescence. The harms of climate change will be more significant for the global poor. Poor countries will suffer the bulk of the damages from climate change, and while adaptation, wealth, and technology may influence distributional consequences, one reason (though not the only reason) poor countries are so vulnerable is their location (e.g., countries in low altitudes start with very high temperatures) (Mendelsohn et al., 2006). An assessment of climate change impacts at the household level also reveals the unequal distributional impacts of climate change for poverty and for poor people, such as the impact of climate change on agricultural productivity and prices, food prices, natural disasters, labor productivity and child stunting, malaria and diarrhea (Hallegatte & Rozenberg, 2017).

Like the unequal distributional impacts climate change has on lower socioeconomic status (SES), the biology of aging also has unequal distributional impacts related to socioeconomic status. Lower SES is related to accelerated aging across a broad range of functional abilities and phenotypes independently of the presence of health conditions and social circumstances impinge on multiple aspects of aging (Steptoe & Zaninotto, 2020). While all humans chronologically age at the same rate, there is substantive variation in the rate of biological aging. This variation is influenced not only by differences in life experiences related to poverty (e.g., smoking, education, diet), but also to individual differences in genetic inheritance and cellular biology. Accelerated aging syndromes like Hutchinson–Gilford progeria syndrome (HGPS) are the most vivid illustration of this point (Ashapkin et al., 2019). Progeria is a rare genetic disorder with an average life expectancy of 13–14 years. But disparities in the pace of biological aging among midlife adults has also been found (Elliott et al., 2021).

The study of the (rare) cohort of humans with exceptional longevity (centenarians (age ≥ 100) and supercentenarians (age ≥ 110)) may reveal the biological secrets behind their decelerated biological aging. Such knowledge can help accelerate the development of gerotherapeutics that enable the average person to enjoy an increased healthspan and compression of frailty, disease, and disability in late life. This aspiration is even more pressing in a warming world because older adults, especially those of low socioeconomic status or belonging to ethnic minority groups, bear a disproportionate share of the health hazards from cardiovascular risk factors intensified from air pollution, heat waves, extreme weather events, etc. (Chang et al., 2022).

## 4 INTERGENERATIONAL CONFLICT AND AGEISM

Despite the disproportionate health burdens older persons face from climate change, older persons are often characterized as those most responsible for climate change, and this can exacerbate attitudes of ageism which stifle geroscience advocacy and thus the prospect that gerotherapeutics will be construed as a pressing public health imperative in a climate‐burdened future. Unfortunately, the problem of climate change is typically discussed as a problem of intergenerational well‐being (Sachs 2015). Many climate scientists and activists deploy findings from social psychology concerning how best to promote pro‐environmental behavior, such as the finding that an increase in awareness of individual responsibility for global warming promotes such behavior (Boto‐García & Bucciol 2020). Climate change anxiety among the young (Wu et al., 2020), coupled with a strong emphasis on personal responsibility for climate change, may actually increase ageism. Possibly blaming others (e.g., holding prescriptive ageist views toward older people) serves as an incentive to take climate change action (Ayalon & Roy 2023). In 2019, the phrase “OK, Boomer” went viral on social media platforms, and became a slogan for “Millennials” who felt “Baby Boomers” were out‐of‐touch with modern problems such as global climate change (Meisner, 2021).

To undercut such sentiments and lines of reasoning, it is important to emphasize how *past* public health interventions have contributed to population growth, and thus, indirectly, to the increase in greenhouse gas emissions. However, this does not mean such public health interventions were not morally laudable. They certainly were. Saving the young from the mortality risks posed by infectious diseases, like tuberculosis, small pox, polio, typhoid fever, measles, mumps, and rubella, were, all‐things‐considered, not only morally permissible, they were *morally obligatory*. The fact that this contributed to an increase in global population, which thus resulted in increases in the emission of greenhouse gases that have contributed to climate change, does not undermine these claims. The young have a *right* to health, and this right demands mitigating the most prevalent morbidity and mortality risks (even if doing so, as we now know, meant an increase in GhGs). Innovations in energy technologies, coupled with alterations in consumption attitudes, means that the causal connection between population size and greenhouse gas emissions need not follow the same trajectory in the decades to come that it did in the past century and half.

The same logic should also be applied to *future* public health interventions that may promote healthy aging (which would increase the healthspan and, as an inadvertent by‐product of doing so, likely increase lifespan). Older persons also have a right to health. The right to health that the young have does not dissipate as they chronologically age, it is a “human right.” The preamble to the Constitution of the World Health Organization defines “health” as follows: “Health is a state of complete physical, mental and social well‐being and not merely the absence of disease or infirmity” (World Health Organization, 2006). The Constitution goes on to affirm that “the enjoyment of the highest attainable standard of health is one of the fundamental rights of every human being without distinction of race, religion, political belief, economic or social condition.” Public health is concerned with the opportunities for health throughout the human lifespan.

Furthermore, the intergenerational conflict often stoked by some climate activists is also empirically inaccurate, as well as ethically dubious. The young of today will be the older generation of tomorrow when both the worsening climate change impacts and potential health benefits of gerotherapeutics may be realized. Aging is a universal phenomenon and the adverse health consequences of senescence are not limited to just the most advanced ages of the lifespan. Slowing biological aging would improve the quality of life of adults at all ages, including potentially the reproductive longevity and maternal health of women (Farrelly, 2023; Llarena & Hine, 2021). The majority of the youth of today are expected to survive into late life and die, predominately, from the degenerative diseases of aging. They will be even more vulnerable in the future because global warming exacerbates many of the health risks of aging.

To combat the ageism that is often implicit, and sometimes explicit, in climate change activism, geroscience must be construed as part of the solution to, versus a contributing factor to the problems of, climate change. Concerns with population size do not warrant entertaining the prospect of forfeiting or stifling future public health interventions any more than they warrant considering forfeiting the existing public health interventions (e.g., sanitation, vaccinations, improvements in nutrition) that have increased life expectancy at birth for a baby in the world to age 73 (World Health Organization, 2021).

## 5 CONCLUSION

Public communication about science occurs along a spectrum between science (“honesty”) and advocacy (“effective”) (Donner, 2014). With respect to the impact of climate change on human longevity, “honesty” may indicate that inaction on climate change could reduce life expectancy at birth by 0.24 years for the average European country (Hauer & Santos‐Lozada, 2021). However, to spur more climate change action, the more “effective” messaging many activists (e.g., Extinction Rebellion and Sunrise Movement), scientists, and even prominent political leaders (e.g., President Joe Biden at the April 2021 Leaders Summit on Climate) invoke narratives about the existential dimension of climate change (Huggel et al., 2022). How climate scientists communicate the risks of climate change will impact how other areas of science, like geroscience, are perceived by the general public and political leaders. What would be the point of investing in the science that might extend human longevity (healthy or otherwise) if climate change will eradicate all human life by 2100? Having a *Declinist Worldview* (e.g., “Modern civilization has reached its peak and is in decline”) is negatively associated with support for life extension (Dragojlovic, 2013).

Rather than conceptualizing the distributional effects of an applied gerontological intervention as something that would only benefit the health of persons in late life (e.g., increasing lifespan), and climate change as something that only imposes health and economic risks on younger generations, we must acknowledge the reality is that aging and climate change are intricately connected. Not only are older persons at higher risk for climate change mortality, but the health of the environments we inhabit (including planetary health) influence aging and the healthspan. Rate (of aging) control would improve the quality of life of adults at all ages. Finally, the economic benefits (Goldman et al., 2013; Olshansky, 2016) of slowing aging can help countries, especially lower and middle income countries, ensure they can afford to invest more in adaptation for climate change. Healthier older populations mean savings on healthcare expenditures, a more productive workforce, human capital development (Rowe, 2015), etc.

By highlighting the complementary aspirations of climate science and geroscience, geroscience advocacy can transcend the narratives of intergenerational conflict typical of climate change activism. The interdependence of climate change and the biology of aging can inspire instead a narrative of *cohesion* (Rowe, 2015) because humanity has a shared interest in both planetary health and healthy aging. The health prospects of everyone will be influenced by the environments in which we live, as well as by the mechanisms and rate of biological aging.

This Perspective has argued that, when it comes to tackling the dual public health predicaments of climate change and healthy aging, advocating for rate (of aging) control can be both an “honest” and “effective” way of getting the general public, policy makers, and other scientists to eschew ageism and the stoking of intergenerational conflict and unsubstantiated catastrophic claims about the future health risks posed by climate change. It is much more fruitful and productive to draw attention to the empirical reality that the biology of aging is intricately connected to some of the most serious health risks posed by climate change. And many of the goals of climate science and geroscience are complementary, such as the aspirations to promote health across the lifespan, redress health disparities, and improve the economic prospects of current and future generations.

## AUTHOR CONTRIBUTIONS

Colin Farrelly is the sole author and was responsible for writing all of the article.

## CONFLICT OF INTEREST STATEMENT

None to declare.

## Data Availability

Data sharing is not applicable to this article as no new data were created or analyzed in this study.
